# Supplementary figures and images for: Lack of the IFN-γ signal leads to lethal Orientia tsutsugamushi infection in mice with skin eschar lesions
Source: PLoS Pathog. 2024 May 14;20(5):e1012020. doi: 10.1371/journal.ppat.1012020 (PMC11125519; doi:10.1371/journal.ppat.1012020)

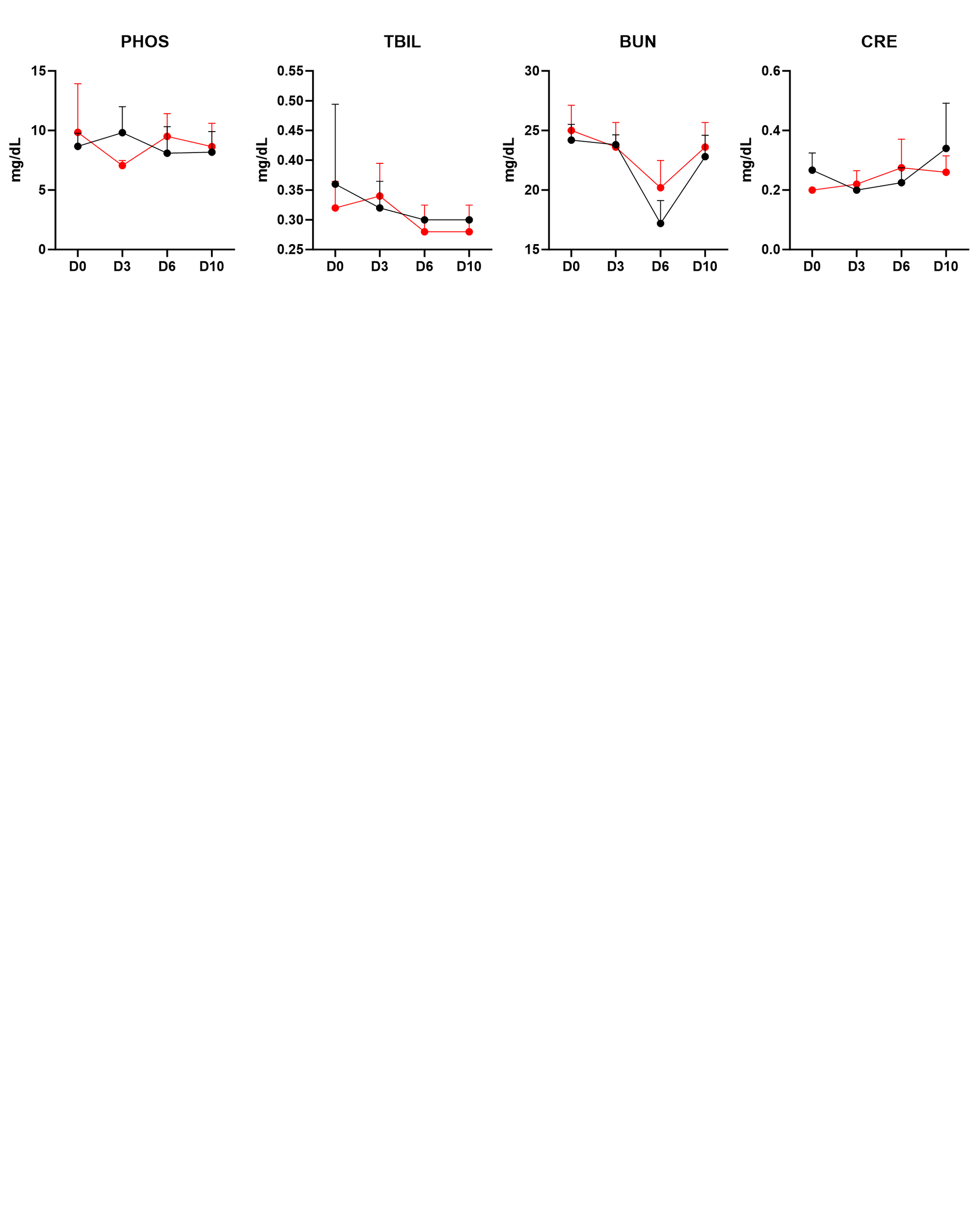

Supplement: S1 Fig — WT and Ifngr1-/- (n = 5/group) were i.d. infected with Ot Karp strain (3×10^3^ FFU) on the flank. The mouse serum chemistry profile was generated by using VetScan Comprehensive Diagnostic Profile reagent rotor. The parameters include creatinine (CRE), phosphorus (PHOS), total bilirubin (TBIL), and urea nitrogen (BUN). The values are shown as mean ± SD from single experiments and are representative of two independent experiments. Two-way ANOVA and Šídák’s multiple comparisons test were used for statistical analysis. (TIF) [file ppat.1012020.s001.tif]

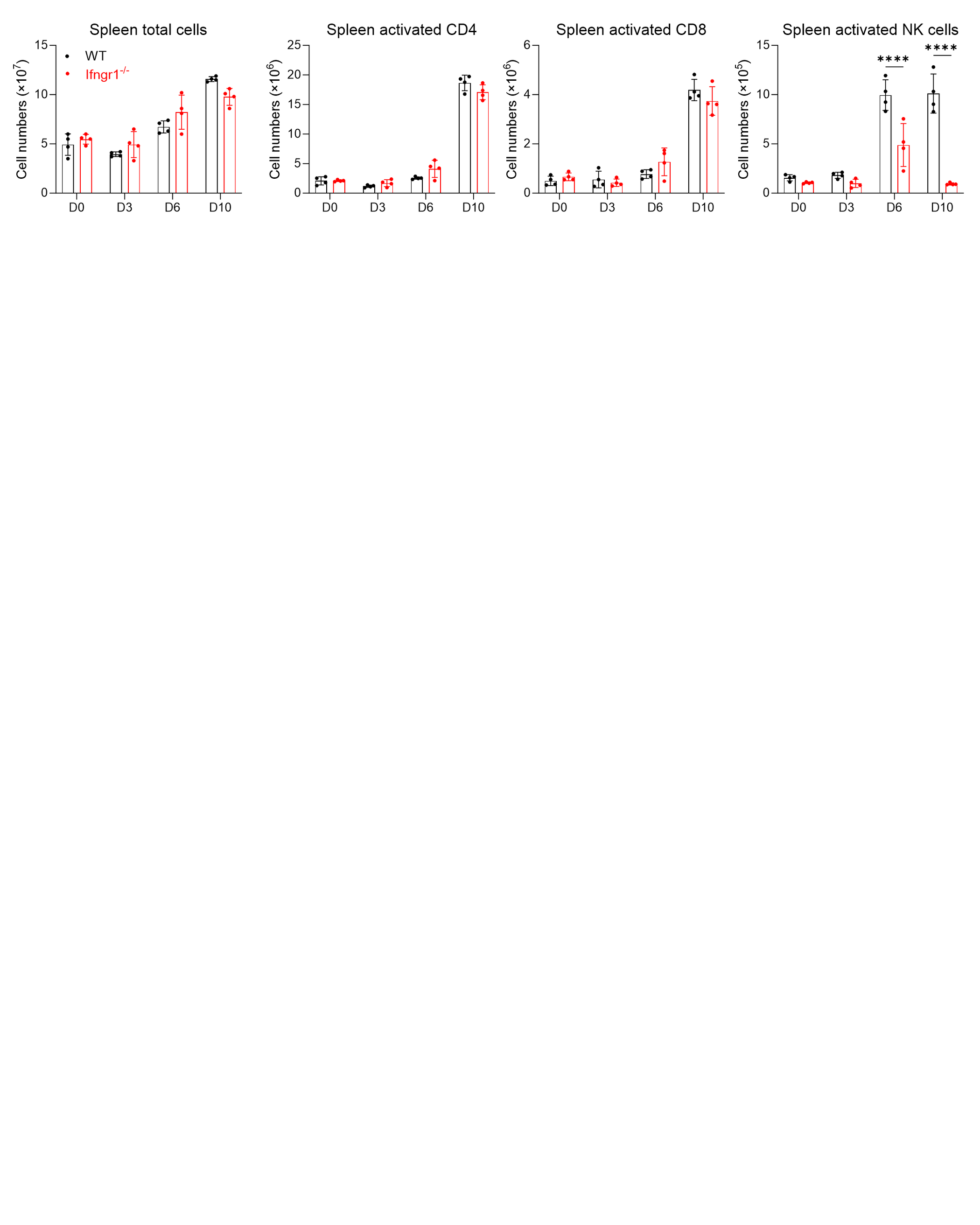

Supplement: S2 Fig — WT and Ifngr1-/- mice (n = 4/group) were i.d. infected with Ot Karp (3×10^3^ FFU). Mouse spleens were collected for preparation of single cell suspensions at days 3, 6 and 10 p.i.. Mock-infected mice were used at day 0. Cell samples were acquired by flow cytometry and data were analyzed by using Flowjo. Gating strategy is shown in Fig 3. Dead cells and doublets were first excluded by live/dead fixable dye and FSC-H vs FSC-W/SSC-H vs SSC-W, respectively. The live and single cells were gated for activated CD4 T cells (CD3+CD4+CD44+CD62L-), activated CD8 T cells (CD3+CD8+CD44+CD62L-), and activated NK cells (CD3-NK1.1+CD69+). Cell numbers are presented as mean ± SD from single experiments and are representative of two independent experiments. Two-way ANOVA was used for statistical analysis. Šídák’s multiple comparisons test was used for multiple comparisons between WT B6 and Ifngr1-/- mice at each time. ****, p < 0.0001. (TIF) [file ppat.1012020.s002.tif]

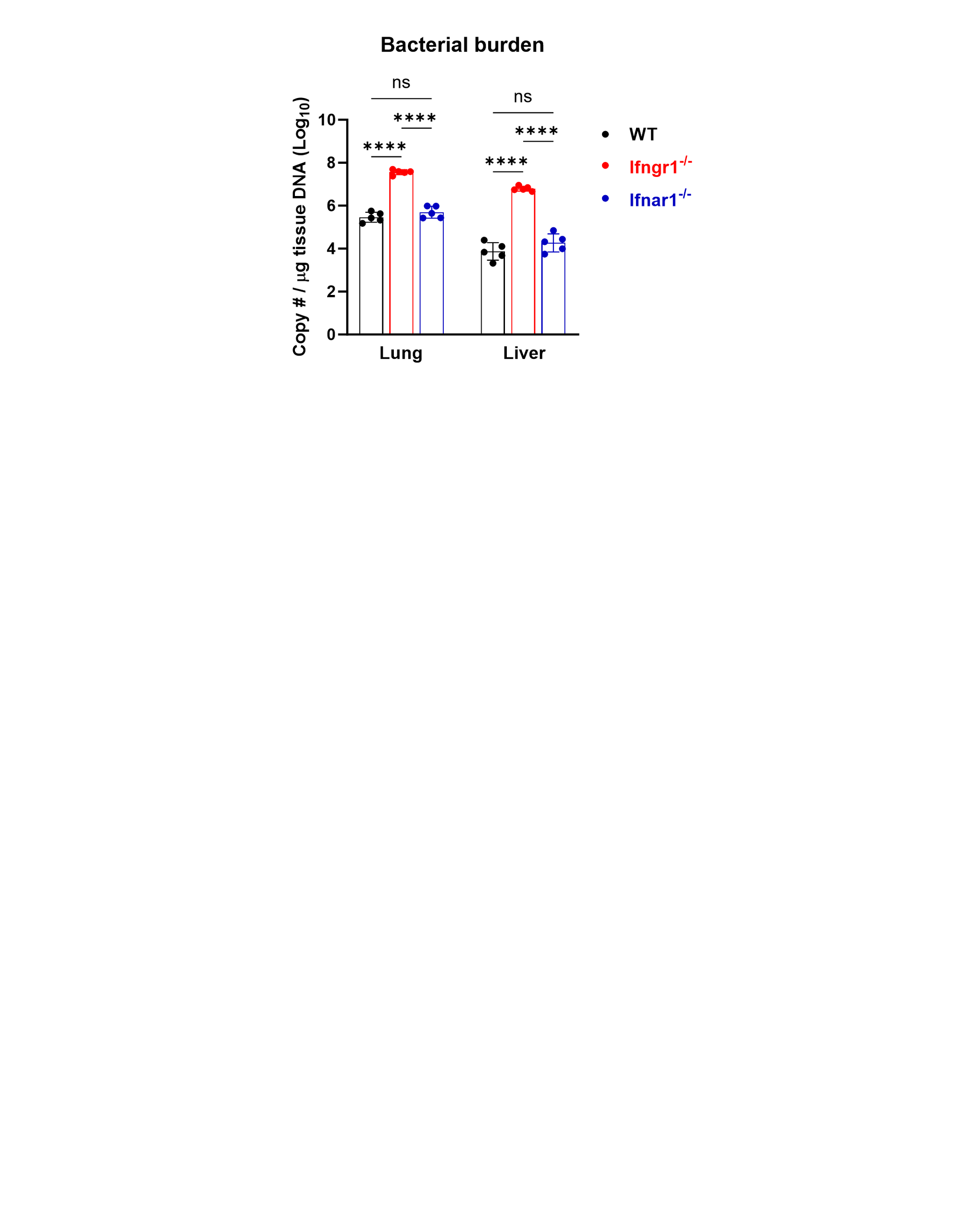

Supplement: S3 Fig — WT, Ifngr1-/- and Ifnar1-/- mice (n = 5/group) were i.d. infected with Ot Karp (3×10^3^ FFU). Lung and liver tissues were harvested on day 10 p.i., followed by the measurement of bacterial burdens via qPCR. The values are shown as mean ± SD from single experiments and are representative of two independent experiments. A one-way ANOVA statistical analysis with a Tukey’s multiple comparisons test was used for multiple comparisons. ****, p < 0.0001; ns, no significance. (TIF) [file ppat.1012020.s003.tif]

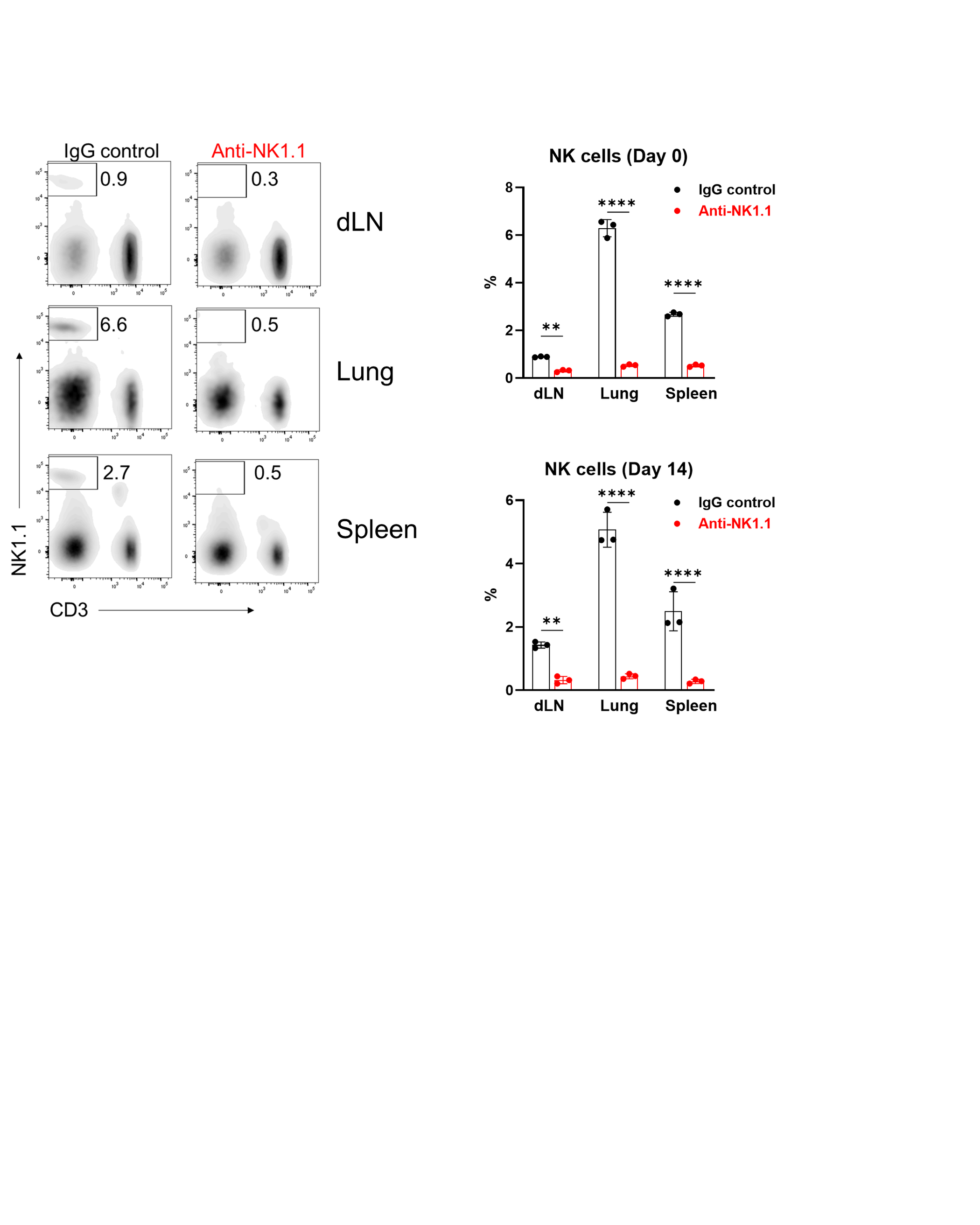

Supplement: S4 Fig — B6 mice (n = 5-6/group) were i.p. treated with either IgG control or anti-NK1.1 neutralizing antibody (200 μg/mouse) on days -3 and -1 and were euthanized on days 0 and 14 p.i.. Single-cell suspensions were prepared by using the dLN, lungs, and spleen, followed by flow cytometric analysis. The representative images of flow cytometry on day 0 were shown. The cell percentages are shown as mean ± SD. Two-tails student t-test was used for comparisons between IgG control and anti-NK1.1 antibody treated samples. **, p < 0.01; ****, p < 0.0001. (TIF) [file ppat.1012020.s004.tif]

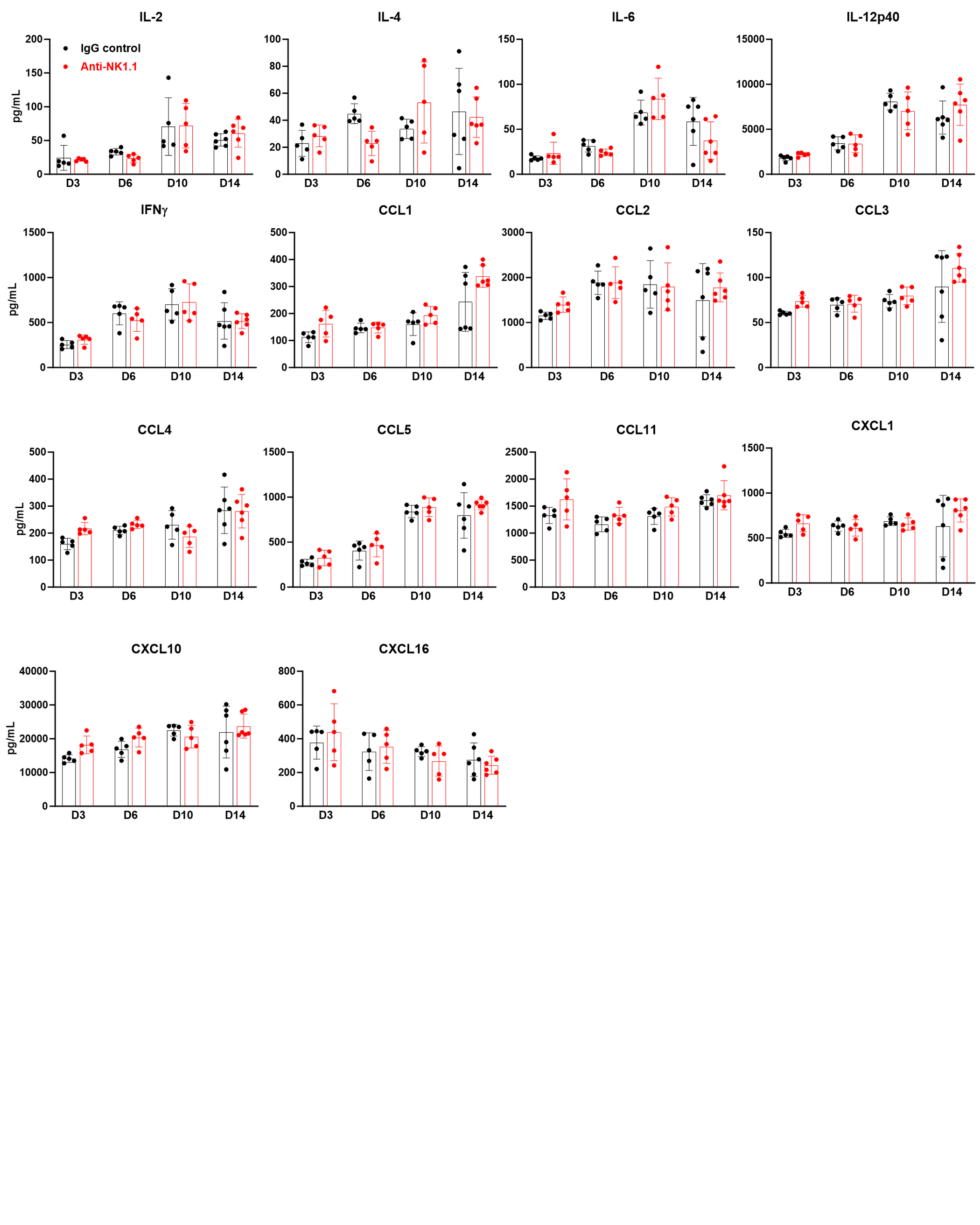

Supplement: S5 Fig — B6 mice (5-6/group) were i.d. infected with Ot Karp (3×10^3^ FFU) and were i.p. treated with either IgG control or anti-NK1.1 neutralizing antibody (200 μg/mouse/treatment) every other day starting from 3 day prior to infection. Mice were euthanized at days 3, 6, 10, and 14 p.i. for tissue/blood harvest. Mouse serum was assessed for by Bio-Plex assay. The values are shown as mean ± SD from single experiments and are representative of two independent experiments. Two-way ANOVA was used for statistical analysis. Šídák’s multiple comparisons test was used for multiple comparisons between IgG control and anti-NK1.1 antibody treated mice at each time. (TIF) [file ppat.1012020.s005.tif]
